# Cryptotanshinone Attenuates Airway Remodeling by Inhibiting Crosstalk Between Tumor Necrosis Factor-Like Weak Inducer of Apoptosis and Transforming Growth Factor Beta 1 Signaling Pathways in Asthma

**DOI:** 10.3389/fphar.2019.01338

**Published:** 2019-11-11

**Authors:** Chongyang Wang, Mingyu Zheng, Yunho Choi, Jingzhi Jiang, Li Li, Junfeng Li, Chang Xu, Zhemin Xian, Yan Li, Hongmei Piao, Liangchang Li, Guanghai Yan

**Affiliations:** ^1^Department of Anatomy, Histology and Embryology, Medical College, Yanbian University, Yanji, China; ^2^Jilin Key Laboratory of Anaphylactic Disease, Yanbian University, Yanji, China; ^3^College of Pharmacy, Yanbian University, Yanji, China; ^4^Department of Anatomy, Medical School, Institute for Medical Sciences, Chonbuk National University, Jeonju, South Korea; ^5^Department of Respiratory Medicine, Affiliated Hospital of Yanbian University, Yanji, China

**Keywords:** cryptotanshinone, airway remodeling, tumor necrosis factor-like weak inducer of apoptosis, transforming growth factor beta 1, STAT3

## Abstract

The study is to investigate the effect of cryptotanshinone (CTS) on airway remodeling and the possible mechanism. Male BALB/c mice were pretreated with CTS or dexamethasone 30 min before nebulized inhalation of ovalbumin (OVA). CTS significantly inhibited OVA-induced increases of eosinophils and neutrophils infiltration of bronchoalveolar lavage fluids (BALFs), reduced airway resistance in asthmatic mice, decreased the accumulation of inflammatory cells, the hyperplasia of goblet cells and the deposition of collagen in asthmatic mice lung tissue, as well as markedly attenuated the leakage of inflammatory cells and the level of OVA-specific immunoglobulin E in BALFs. CTS also inhibited the expressions of alpha-smooth muscle actin, tumor necrosis factor-like weak inducer of apoptosis (TWEAK), Fn14, transforming growth factor (TGF)-β1, Smad4, and phosphorylation of Smad2/3 and STAT3 (Tyr705). In comparison to TWEAK inhibitor or TWEAK small interfering RNA (siRNA), which were used to inhibit TWEAK/STAT3 signaling pathways, CTS caused a similar effect as them on airway remodeling. Additionally, CTS also played a similar role as the TGF-β1 inhibitor or TGF-β1 siRNA in TGF-β1/STAT3 signaling pathways in airway remodeling. The anti-inflammatory effects of CTS against OVA-induced airway remodeling may be through inhibiting STAT3, which further suppresses TWEAK and TGF-β1 signaling cross talk in asthma. CTS may be a promising therapeutic reagent for asthma treatment.

## Introduction

Airway hyperresponsiveness (AHR), inflammation, and remodeling are the major characteristics of asthma; among them, airway remodeling, which occurs at a very early stage of asthma in parallel with inflammation, has gained attention (Capra and Rovati, 2014). The pathological changes in airway remodeling include epithelial damage, subepithelial fibrosis, mucus gland hyperplasia, increased smooth muscle mall, and vascular hyperplasia. Airway remodeling can induce excessive narrowing of the airway and cause irreversible airway obstruction, eventually, intractable asthma is developed. Studies have shown that airway remodeling can occur independent of inflammation or pulmonary function changes (Stenmark et al., 2015). Great efforts have been directed toward the management of airway remodeling; however, few strategies have been proven to be effective. Thus, there is an urgent need to develop new therapeutic options for airway remodeling in asthma (Liu et al., 2015).

Tumor necrosis factor-like weak inducer of apoptosis (TWEAK), is expressed in various cell types, including monocytes, macrophages, dendritic cells, T cells, and NK cells (Burkly et al., 2007). TWEAK acts through fibroblast growth factor-inducible 14 (Fn14), its highly inducible receptor, and plays a multifunctional role by regulating diverse cellular responses, including pro-inflammatory activity, cell growth, angiogenesis, progenitor cell biology, and even cell death (Burkly et al., 2007). In addition, TWEAK is significantly elevated in inflammatory diseases (Schwartz et al., 2006; Kamijo et al., 2008; Serafini et al., 2008) and cancer (Winkles, 2008). Furthermore, it has been reported that TWEAK/Fn14/NF-κB pathway plays an important role in asthma airway inflammation of asthma (Kim et al., 2018; Zhu and Zhang, 2018), and can induce proliferation and migration of human airway smooth muscle cells (ASMCs) (Zhu and Zhang, 2018). Thus, it is likely that TWEAK/Fn14 is associated with the airway remodeling in asthma.

Transforming growth factor (TGF)-β1, a member of the TGF superfamily, can regulate growth, differentiation, and immune functions of cells. Several reports have suggested that TGF-β1 plays a crucial role in airway remodeling and proliferation of ASMCs (Chen and Khalil, 2006; Al-Muhsen et al., 2011; Al-Alawi et al., 2014). The hyperplasia and hypertrophy of ASMCs induced by TGF-β1 is probably attributed to the generation of intracellular reactive oxygen species (Sturrock et al., 2007). Previous studies (Liu S. et al., 2018; Wang Q. et al., 2018) have shown that TGF-β1 exerts its biological function by Smad protein family. Moreover, it has been reported that TGF-β1/Smad and NF-κB signaling pathway is involved in fibrosis and inflammation (Zheng et al., 2018). Taken together, it is assumed that TGF-β1/Smad signaling pathway may be associated with airway remodeling in asthma.

Signal transducers and activators of transcription (STAT) proteins can transduce extracellular signals and regulate transcription of target genes (Shin et al., 2009). Among the STATs, STAT3 is most closely related with tumorigenesis (Aggarwal et al., 2006) and is constitutively activated and overexpressed in various tumor types such as breast carcinoma, prostate cancer, melanoma, multiple myeloma, and leukemia (Liu et al., 2017; Pathak et al., 2007; Wang Z. et al., 2018). STAT3 can be activated by many factors and promote inflammation (Vukelic et al., 2018; Zegeye et al., 2018). STAT3 is also regulated by TWEAK and TGF-β1 signaling pathway (Chaturvedi and Misra, 2018; Hao et al., 2018). Thereby, STAT3 may play a role in asthmatic airway remodeling.

*Salvia miltiorrhiza* Bunge is a well-known traditional medicine used for the treatment of allergic diseases (Chang et al., 2018). A study has shown that *S. miltiorrhiza* Bunge significantly inhibited endothelial hyperplasia and proliferation (Chen et al., 2001). Cryptotanshinone (CTS) is a main ingredient of *S. miltiorrhiza* Bunge. It has been demonstrated that CTS exhibits anti-inflammatory, anti-oxidant (Liu C. et al., 2018), anti-proliferative (Hamzaoui et al., 2001), anti-cancer (Shen et al., 2017; Teng et al., 2018) and anti-fibrotic activities in various disorders (Wang et al., 2013). Furthermore, it has been revealed that CTS suppresses renal fibrosis and epithelial transdifferentiation in obstructive nephropathy by inhibiting TGF-β1/Smad3 signaling pathway (Wang W. et al., 2018). Importantly, CTS is an inhibitor of STAT3. We hypothesize that the attenuation of airway remodeling by CTS might be through the suppression of STAT3 and inhibition of TWEAK/TGF-β1 signaling cross talk.

Herein, we investigated the effect of CTS on airway remodeling and the possible mechanism. The results showed that CTS could ameliorate airway remodeling by suppression of TWEAK and TGF-β1 signaling cross-talk. This study demonstrated the action mechanism of CTS, suggesting it may be a promising therapeutic reagent for asthma treatment.

## Materials and Methods

### Animals

Male C57BL/6 mice (8–10 weeks old; weight 20–22 g) were obtained from the HOUSE section of Yanbian University Health Science Center (Yanji, China). All mice were acclimatized at 22 ± 2°C, 50–60% of relative humidity, and 12-h light-dark cycle. The experiment procedures were approved by the Institutional Animal Care and Use Committee of Yanbian University (resolution number, 201501022).

### Animal Grouping and Treatment

**Figure 1:**
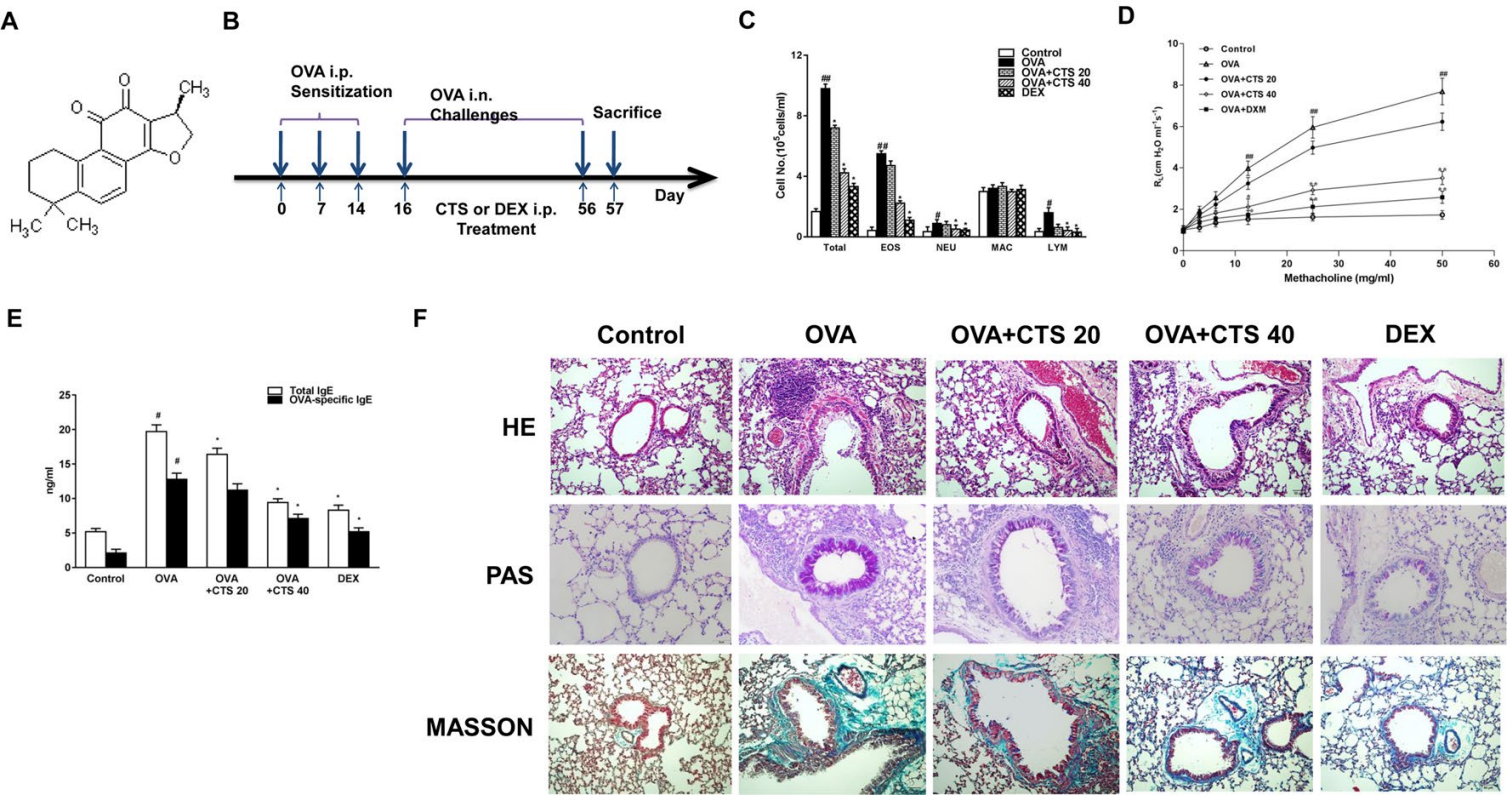
The effect of CTS on the inflammation and airway hyperresponsiveness of the OVA-inhaled mice. **(A)** Chemical structure of CTS. **(B)** A schematic diagram of the experimental protocol. The mice were sensitized on day 0, 7, and 14 by intraperitoneal injection of 20 µg ovalbumin (OVA) emulsified in 2 mg of aluminum hydroxide gel in a total volume of 200 µl. These sensitized mice were exposed to aerosolized 5% OVA in sterile saline for three times (30 min each time) a week for 8 weeks. The mice were placed in chambers connected to an ultrasonic nebulizer to obtain a whole-body inhalation. CTS and dexamethasone (DEX) were administered through intraperitoneal injection at 30 min before nebulization. The control mice were sensitized and challenged with phosphate buffered saline (PBS) using the same protocol. **(C)** The numbers of total and separate cellular components of bronchoalveolar lavage fluids (BALFs) were counted. EOS, eosinophil; NEU, neutrophil; MAC, macrophage; LYM, lymphocyte. **(D)** Lung resistance (RL) measurement was performed at 24 h after the last challenge in mice. **(E)** The effect of CTS on total and OVA-specific immunoglobulin E (IgE) levels in the BALFs of OVA-inhaled mice. The levels of total and OVA-specific IgE were quantified by ELISA. **(F)** Lung sections were stained with hematoxylin-eosin (HE) to analyze the infiltration of inflammatory cells; periodic acid-Schiff (PAS) to assess goblet cell hyperplasia; and Masson’s trichrome to evaluate the subepithelial deposition of collagen and fibrosis. Magnification was 200×. Scale bars indicate 50 μm. The data were expressed as mean ± SEM (standard error of mean, n = 8). #P < 0.05, ##P < 0.01 vs. untreated mice; *P < 0.05, **P < 0.01 vs. OVA-treated mice. Sampling was performed 48 h after the last challenge in mice. PBS-inhaled mice were administered with PBS (control); OVA-inhaled mice were administered with OVA dissolved in PBS; in the treatment group, OVA-inhaled mice were respectively administered with 20 and 40 mg/kg cryptotanshinone (CTS) (OVA+CTS 20 and OVA+CTS 40 groups); and OVA-inhaled mice administered with dexamethasone (OVA+DEX) was use as a positive control.

Mice were randomly separated into the following five groups: control group (n = 8), OVA group (ovalbumin, Sigma Co., St. Louis, MO, USA; n = 8), OVA+CTS 20 (CTS 20 mg/kg; Abcam, ab120666; Purity > 97% with chemical structure shown in **Figure 1A**; n = 8), OVA+CTS 40 (CTS 40 mg/kg; n = 8), and OVA+DEX (dexamethasone 1 mg/kg; Sigma Co., St. Louis, MO, USA; n = 8). The mice were sensitized on day 0, 7, and 14 by intraperitoneal injection of 20 µg OVA emulsified in 2 mg of aluminum hydroxide gel (InvivoGen, San Diego, CA, USA) in a total volume of 200 µl. These sensitized mice were exposed to aerosolized 5% OVA in sterile saline for three times (30 min each time) a week for 8 weeks beginning on day 16. The mice were placed in chambers (18×14×8 cm) connected to an ultrasonic nebulizer (NE-U11B; Omron Corp., Tokyo, Japan) to obtain a whole-body inhalation. CTS and DEX were administered through intraperitoneal injection at 30 min before nebulization (**Figure 1B**). Control mice were sensitized and challenged with phosphate buffered saline (PBS) using the same protocol. The mice were sacrificed at 48 h after the last challenge, and bronchoalveolar lavage fluid (BALF) and lung tissues were collected for analysis.

### Assessment of Airway Responsiveness

Twenty-four hours after the last challenge, lung resistance measurement (SCIREQ, Montreal, Canada) was used to estimate AHR. Briefly, mice were anesthetized by pentobarbital sodium (100 mg/kg) and entered a deep anesthesia state. The trachea was exposed and a cannula was inserted. After administration with grading doses of aerosol methacholine (3.125, 6.25, 12.5, 25, and 50 mg/ml), lung resistance of mice was marked to quantitatively assess AHR.

### Bronchoalveolar Lavage Fluids Collection and Differential Cell Count

Immediately following the assessment of airway responsiveness, the mice were anesthetized with ethyl ether and the tracheas were cannulated while gently massaging the thorax. The lungs were lavaged with 0.8 ml of PBS. The BALF samples were collected and the number of total cells in a 0.05 ml aliquot was counted using a hemocytometer (Baxter Diagnostics, Deerfield, IL, USA). The remaining samples were centrifuged. The supernatants were stored at −80°C, while the cell pellets were resuspended in PBS. The BAL cells were smeared and stained with Diff-Quik solution (International Reagents, Kobe, Japan). The cell differentials were then enumerated based on the cell morphology and staining profile.

### Lung Histology

After BALF samples were collected, the lungs were inflated with 4% paraformaldehyde and embedded in paraffin. The tissues were cut into 5 µm sections. H&E staining was performed to analyze the morphological changes. Periodic acid-Schiff (PAS) staining was also conducted to assess airway global cells and mucus production. The percentage of PAS staining-positive cells in the airway epithelium was quantified. Masson’s trichrome staining was performed to visualize collagen deposition and fibrosis. Quantitative assessment was carried out by calculating the percentage of Masson trichrome staining positive area in bronchial airway.

### Immunohistochemistry

Immunohistochemical staining was performed to examine expression and distribution of proliferating cell nuclear antigen (PCNA), alpha-smooth muscle actin (α-SMA), TWEAK, and TGF-β1. Briefly, the sections were incubated with primary antibodies against PCNA (1:100 dilution, Abcam, ab92552, Cambridge, MA, USA), α-SAM (1:50 dilution, Affinity, AF1032, china), TWEAK (1:100 dilution, Abcam, ab199419, Cambridge, MA, USA), or TGF-β1 (1:50 dilution, Abcam, ab31013, Cambridge, MA, USA) followed by the incubation with peroxidase-conjugated secondary antibodies (goat anti-rabbit IgG-HRP, 1:500 dilution, Abcam, ab205718) (Bao et al., 2009; Provoost et al., 2016). After immunostaining, the slides were counterstained with hematoxylin and then mounted with an aqueous mounting medium (Thermo Shandon Immu-Mount, Pittsburgh, PA, USA). Under 200× magnification, five fields of each section were randomly selected and photographed (Eclipse E600, Nikon, Japan).

### Enzyme-Linked Immunosorbent Assay

The levels of total and OVA-specific immunoglobulin E (IgE), interleukin 4 (IL-4), IL-5, IL-13, tumor necrosis factor alpha (TNF-α), IL-1β, intercellular cell adhesion molecule 1 (ICAM-1), and vascular CAM-1 (VCAM-1) in BALF were determined using the mouse ELISA Kits (R&D Systems, Minneapolis, MN, USA). The sensitivity for IL-4, IL-5, IL-13, TNF-α, and IL-1β is 2.0 pg/ml. The lower limits of detection for ICAM-1 and VCAM-1 are 1.5 pg/ml and 2 ng/ml, respectively.

### Western Blot

As described previously (Choi et al., 2013), freshly isolated lung tissues and whole cells were homogenized and prepared in the presence of protease inhibitors, and the protein concentrations were determined using the Bradford reagent (Bio-Rad, Hercules, CA, USA). Protein samples were separated on sodium dodecyl sulfate polyacrylamide gel electrophoresis and then transferred onto nitrocellulose membranes. Western blot analysis was performed using the polyclonal antibodies against IL-4 (Abcam, ab11524, 1:1,000 dilution), IL-5RA (Abcam, ab104087, 1:500 dilution), IL-13 (Abcam, ab106732, 1:1,000 dilution), TNF-α (Elabscience, ENT4689, China), IL-1β (Elabscience, ENT2322, China), ICAM-1 (Abcam, ab171123, 1:500 dilution), VCAM-1 (Abcam, ab134047, 1:2,000 dilution), cyclin D1 (Abcam, ab134175, 1:10,000 dilution), B-cell lymphoma-extra large (Bcl-xL) (Abcam, ab32370, 1:1,000 dilution), Bax (Abcam, ab199677, 1:1,000 dilution), TWEAK, Fn14 (Cell Signaling Technology, #4403, 1:1,000 dilution, Danvers, MA, USA), TGF-β1, p-Smad2/3 (Abcam, ab63399, 1:1,000 dilution), Smad2/3 (Abcam, ab202445, 1:1,000 dilution), Smad4 (Abcam, ab230815, 1:1,000 dilution), p-STAT3 (Tyr705, Abcam, ab76315), p-STAT3 (Ser727, Cell Signaling Technology, #9134, 1:1,000 dilution), STAT3 (Abcam, ab68153), and β-actin (Cell Signaling Technology, #3700, 1:1,000 dilution). The goat anti-mouse IgG-HRP (Abcam, ab205719, 1:2,000 dilution), goat anti-rat IgG-HRP (Abcam, ab205720, 1:2,000 dilution), and goat anti-rabbit IgG-HRP (Abcam, ab205718, 1:2,000 dilution) secondary antibodies were used. Enhanced chemiluminescent was used for color development. The gray values of the protein bands were analyzed by ImageJ, and the relative protein expression was calculated using β-actin as the internal standard.

### Airway Smooth Muscle Cell Isolation and Cell Culture

ASMCs were isolated from BALB/c mice. The airway tissues were digested with collagenase type I and papain at 37°C for 25 min. They were then treated with Dulbecco’s Modified Eagle Medium (DMEM) (Gibco BRL, Grand Island, NY, USA) to terminate the digestion and centrifuged at 1,000 rpm for 5 min. The supernatant was then removed and the remaining pellet was further digested with 0.25% pancreatin at 37°C for 15 min, followed by digestion termination and centrifugation as the previously step. The ASMCs in the pellet were isolated and cultured incubated at 37°C in DMEM supplemented with 10% fetal bovine serum (Gibco BRL) and 1% penicillin/streptomycin (Gibco BRL) at 37°C.

Recombinant human TWEAK/TGF-β1 (Abcam, ab9968/ab166886) was reconstituted with 0.1% FBS and stored at −20°C. For TWEAK/TGF-β1 stimulation, the cultured media were changed to growth factor and serum free fresh solution with or without recombinant soluble human TGF-β1 (10 ng/ml), or TWEAK (100 ng/ml), or a mixture of TWEAK (10 ng/ml)+TGF-β1 (100 ng/ml). The treatment was performed for 24 h.

### Flow Cytometry

Cell cycle was analyzed using a Cell cycle Analysis Kit (Beyotime, Suzhou, Jiangsu, China) according to the instruction of the kit. The percentage of cells in each phase of the cell cycle was detected by CytoFLEX Flow Cytometry (Beckman Coulter, Inc., CA, USA) and analyzed with CyExpert (Beckman Coulter Inc., CA, USA).

### Immunofluorescence

ASMCs were grown in six-well chambers and fixed with 4% paraformaldehyde for 15 min, permeabilized with 0.5% Triton X-100 (CWBIO, China) and blocked with 5% BSA for 2 h. The cells were incubated with the primary antibodies of TWEAK/Fn14 (Abcam, ab85089, 1:100 dilution), p-Smad2/3, Smad4, and p-STAT3 at 4°C overnight, washed three times with PBS, incubated with Alexa Fluor 488 or Alexa Fluor 568-labeled goat anti-rabbit IgG secondary antibody (Invitrogen) for 2 h, and then observed and photographed by a confocal microscope (Cytation™ 5, BioTek, USA).

### Ribonucleic Acid Interference

ASMCs cells were transfected with TWEAK small interfering RNA (siRNA) (50 nmol/L; s233935; AMBION, Thermo Fisher Scientific, USA), TGF-β1 siRNA (50 nmol/L; s74452; AMBION), and negative control siRNA (50 nmol/L) using Lipofectamine 3000 (Invitrogen, Carlsbad, CA, USA). The primer sequences were as follows: TWEAK siRNA forward, 5′-ACTCTTTCAAGTTCACTGA-3,′ TWEAK siRNA reverse, 5′-TCTTCTTTAACATCCCATCC-3′; TGF-β1 siRNA forward, 5′-GGGCUACCAUGCC AACUUCTT-3,′ TGF-β1 siRNA reverse, 5′-GAAGUUGGCAUGGUAGCCCTT-3.′ To identify whether the genes were silenced completely, PCR was used. After 72 h of transfection, reverse transcription (RT)-PCR and Western blot were used to detect transfection efficiency.

### Reverse Transcription Polymerase Chain Reaction

Reverse transcription PCR was used to detect the expressions of TWEAK, Fn14, TGF-β1, and Smad3 messenger RNA (mRNA). Total RNA was isolated using the RNA Easy Kit (Invitrogen, USA). The complementary DNA was reverse transcribed from 1 µl of total RNA per 25 µl RT reaction with Oligo (dT) 15 primer and the AMV Reverse Transcriptase (Takara, Otsu, Shiga, Japan). The primer sequences were as follows: TWEAK (forward-5′-TCACCCGGGCTGGGCTCTAC-3,′ reverse-5′-CGAGGGAACTGGCC GCAGTG-3′), Fn14 (forward-5′-ACCTGGACAAGTGCATGGACTGC-3,′ reverse-5′-GCGTGAGGCTCCTTTCTGTT-3′), TGF-β1 (forward-5′-CCTTGCCCTCTAC AACCAACAC-3,′ reverse-5′-CTTGCAGGAGCGCACGATC), Smad2/3 (forward-5′-GGCTTCGGCTGGGCTTTC-3,′ reverse-5′-AGTGAGTGGGCGGAGACT-3′), Smad4 (forward-5′-AAGGATCAAAATTGCTTCAGA-3,′ reverse-5′-CAGTCTAA AGGTTGTGGGTC-3′), and GAPDH (forward-5′-CAAGGTCATCCATGACAAC TTTG-3,′ reverse-5′-GTCCACCACCCTGTTGC TGTAG′). The PCR products were subjected to electrophoresis and stained with ethidium bromide. The expression levels of all the transcripts were normalized to that of GAPDH mRNA in the same samples.

### Statistical Analysis

Data were analyzed by one-way analysis of variance (ANOVA) followed by Dunnett’s *post hoc* test using Prism7 (GraphPad Software Inc., San Diego, CA, USA). ImageJ was used to analyze and quantify the images. The data were expressed as mean ± standard error of the mean (SEM). A *P* value less than 0.05 was considered statistically significant.

## Results

### Effect of Cryptotanshinone on the Ovalbumin-Induced Chemotaxis, Airway Hyperresponsiveness, and Lung Histological Structures of the Asthma Model Mice

During asthmatic attack, airway inflammation is marked by increased number of inflammatory cells in the airways and pulmonary subepithelial spaces (Boyce et al., 2009). To examine the effect of CTS on chemotaxis, that is, recruitment of inflammatory cells in BALFs after OVA-sensitized, the total and separate cellular components of BALFs were counted. As shown in **Figure 1C**, the numbers of total cells, eosinophils, neutrophils and lymphocytes in BALFs all increased significantly at 48 h after OVA inhalation compared with control (*P* < 0.05). The numbers of total cells, eosinophils, neutrophils and lymphocytes in BALFs were markedly reduced by high dose CTS or DEX treatment compared to the OVA group (*P* < 0.05). However, in the low dose CTS group, only the number of total cells decreased compared to the OVA group (*P* < 0.05). These results indicate that high dose CTS can decrease inflammatory cells in BALFs after OVA-sensitization, especially eosinophils.

AHR is indicative of chronic inflammation in the airways, which results from the airflow restriction due to mucus hypersecretion and remodeling, leading to airway obstruction (Brannan and Lougheed, 2012). To demonstrate the effect of CTS on airway AHR, airway responsiveness was assessed as the percentage of an increase in response to the increasing doses of methacholine by an invasive measurement of lung resistance. As shown in **Figure 1D**, the lung resistance produced by the administration of methacholine at 12.5, 25, and 50 mg/ml was significantly greater in the OVA-inhaled mice than that in the control mice (*P* < 0.01). The OVA-sensitized mice treated with high dose of CTS and DEX showed a substantial reduction in the methacholine (25 and 50 mg/ml)-induced lung resistance compared with the untreated mice after OVA inhalation (P < 0.01). These results indicate that the administration of CTS reduces OVA-induced AHR in a murine model of asthma.

The histological changes of lung parenchyma were further evaluated. The lungs from the OVA-sensitized mice showed widespread peribronchiolar and perivascular inflammatory cell infiltration compared with control (**Figure 1F**). However, the administration of high dose of CTS or DEX caused an obvious reduction of inflammatory cell infiltration. These results indicate that treatment with high doses of CTS inhibits the infiltration of inflammatory cells and attenuates allergic airway inflammation. We then determined the effect of CTS on mucus hypersecretion. The mice lung sections were stained with PAS and the number of PAS-positive cells was determined. Epithelial goblet cell that was stained positively with PAS in OVA-inhaled mice was substantially increased than that in control mice (**Figure 1F**). This increase was significantly decreased by high dose of CTS or DEX. Meanwhile, the extent of collagen deposition/fibrosis was evaluated by Masson’s trichrome stain. The collagen deposition was profoundly increased over the interstitium of the airway and vessels in the OVA group (**Figure 1F**). This increase in airway collagen deposition and fibrosis was reversed by the treatment with high doses of CTS or DEX. These indicate that CTS can reduce inflammatory cell infiltration, the proliferation of goblet cells, and the extent of fibrosis of the OVA-inhaled mice.

### Cryptotanshinone Decreases the Levels of Inflammatory Cytokines and Inflammatory Response Markers in the Bronchoalveolar Lavage Fluids of Ovalbumin-Inhaled Mice

**Figure 2:**
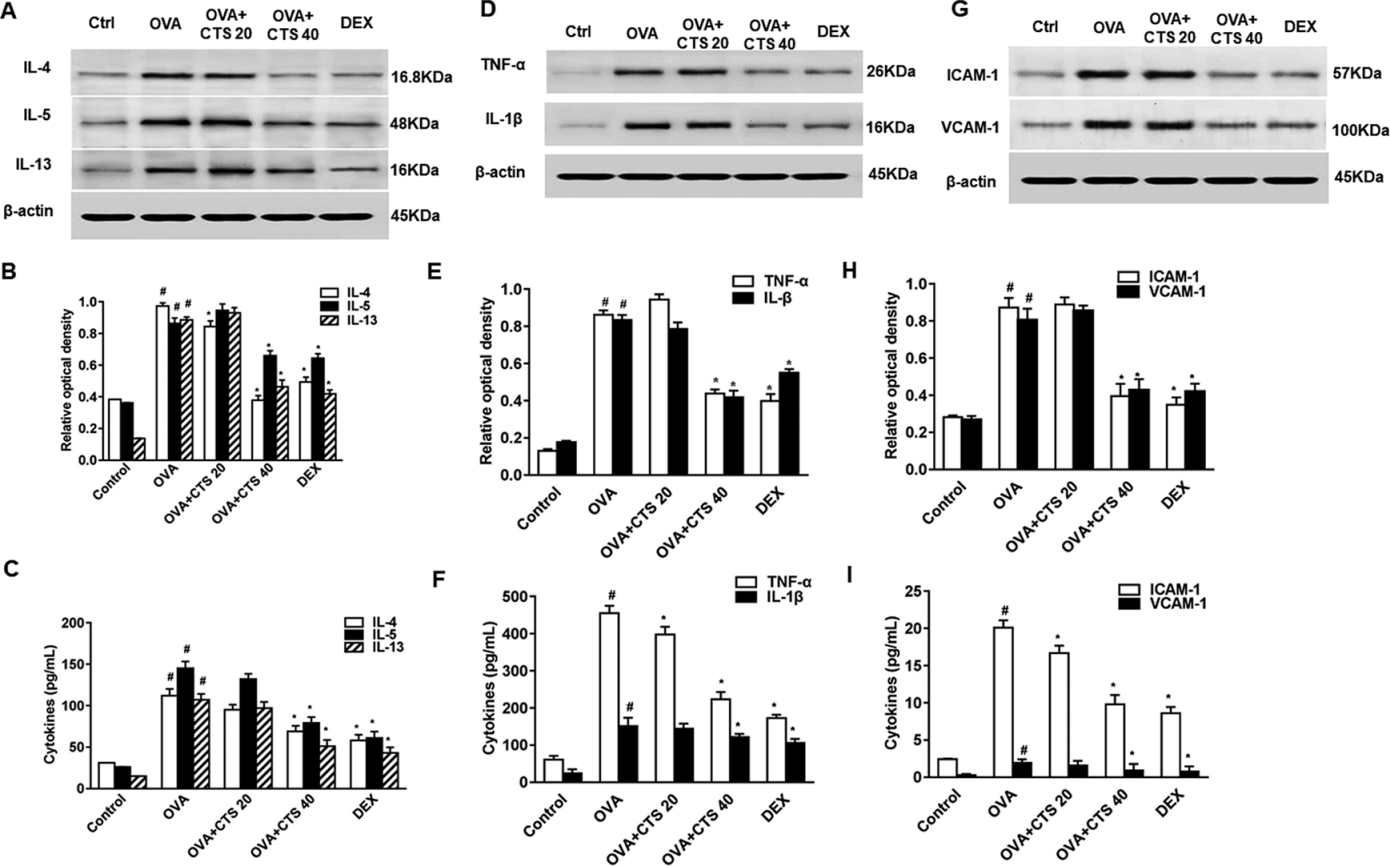
Effect of cryptotanshinone (CTS) on ovalbumin (OVA)-induced cytokines elevation and assessment of immunoglobulin E (IgE). **(A**, **D**, and **G)** The levels of interleukin 4 (IL-4), IL-5, IL-13, IL-1β, tumor necrosis factor alpha, intercellular cell adhesion molecule-1, and vascular cell adhesion molecule 1 in lung tissues were detected by Western blot. **(B**, **E**, and **H)** Densitometric analysis was presented as the relative ratio of each molecule to β-actin. The relative ratio of each molecule in the lung tissues of untreated mice is arbitrarily presented as 1. **(C**, **F**, **I)** The levels of cytokines, total and OVA-specific IgE in bronchoalveolar lavage fluids supernatant from each group were detected by ELISA. Data were shown as mean ± SEM (n = 8). #P < 0.05 vs. untreated mice; *P < 0.05 vs. OVA-treated mice. Control (PBS-inhaled mice administered with PBS), OVA (OVA-inhaled mice administered with PBS), OVA+CTS 20/40 (OVA-inhaled mice administered with 20 mg/kg or 40 mg/kg CTS), and dexamethasone (DEX) (OVA-inhaled mice administered with DEX).

To determine the effect of CTS on inflammatory cytokines in lung tissues and BALFs, Western blot and enzyme-linked immunosorbent assay (ELISA) was performed. Western blot analysis revealed that protein expressions of IL-4, IL-5, IL-13, TNF-α, and IL-1β in lung tissues was significantly upregulated in OVA-inhaled mice compared with control (**Figures 2A, B–E**). The elevated levels of these cytokines were significantly reduced by a high dose of CTS administration (*P* < 0.05), but not the low dose. Consistently, ELISA showed that the levels of IL-4, IL-5, IL-13, TNF-α, and IL-1β in the BALFs were significantly increased in the OVA-inhaled mice. However, these effects were attenuated by a high dose of CTS and DEX treatment (**Figures 2C, F**, *P* < 0.05). However, there was no significant difference between low dose CTS group and OVA group (*P* > 0.05). These indicate that high dose CTS can reduce the levels of inflammatory cytokines and inflammatory response markers in the BALFs of OVA-inhaled mice.

Leukocyte-endothelial adhesion molecules (ICAM-1 and VCAM-1) are important for the recruitment and migration of leukocytes to the inflammation sites (Wegner et al., 1990; Hamzaoui et al., 2001). The effect of CTS on ICAM-1 and VCAM-1 expressions in OVA-sensitized mice were also examined by ELISA and Western blot. ELISA showed that the levels of ICAM-1 and VCAM-1 in BALFs were significantly increased in the OVA-treated mice compared with the levels in the control mice whereas significantly decreased by the administration of a high dose of CTS (*P* < 0.05, **Figure 2I**). However, no significant difference was found between the low dose CTS group and the OVA group (*P* > 0.05). Similarly, Western blot analysis revealed that the protein levels of ICAM-1 and VCAM-1 in lung tissues increased at 48 h after OVA challenge compared with control. The increased levels of these proteins were significantly reduced by the high dose of CTS or DEX (**Figures 2G, H**). These findings indicate that CTS efficiently represses the increase of adhesion molecules in BALFs and lung tissues of the OVA-treated mice.

### Cryptotanshinone Reduces the Level of Immunoglobulin E in the Bronchoalveolar Lavage Fluids of Ovalbumin-Inhaled Mice

A key feature of airway asthma is the high level of IgE. Thus, the total and OVA-specific IgE levels were determined by ELISA. The IgE levels in BALFs were dramatically elevated in the OVA-sensitized mice, compared with the control mice. The administration of high dose of CTS led to a significant reduction in the total and OVA-specific IgE levels, but the low dose of CTS reduced only the total IgE level (*P* < 0.05, **Figure 1E**). This indicates that a certain dose of CTS could reduce the level of IgE in the BALFs of OVA-inhaled mice.

### Cryptotanshinone Suppresses Tumor Necrosis Factor-Like Weak Inducer of Apoptosis, Transforming Growth Factor Beta 1, and STAT3 Activation in Ovalbumin-Inhaled Mice

**Figure 3:**
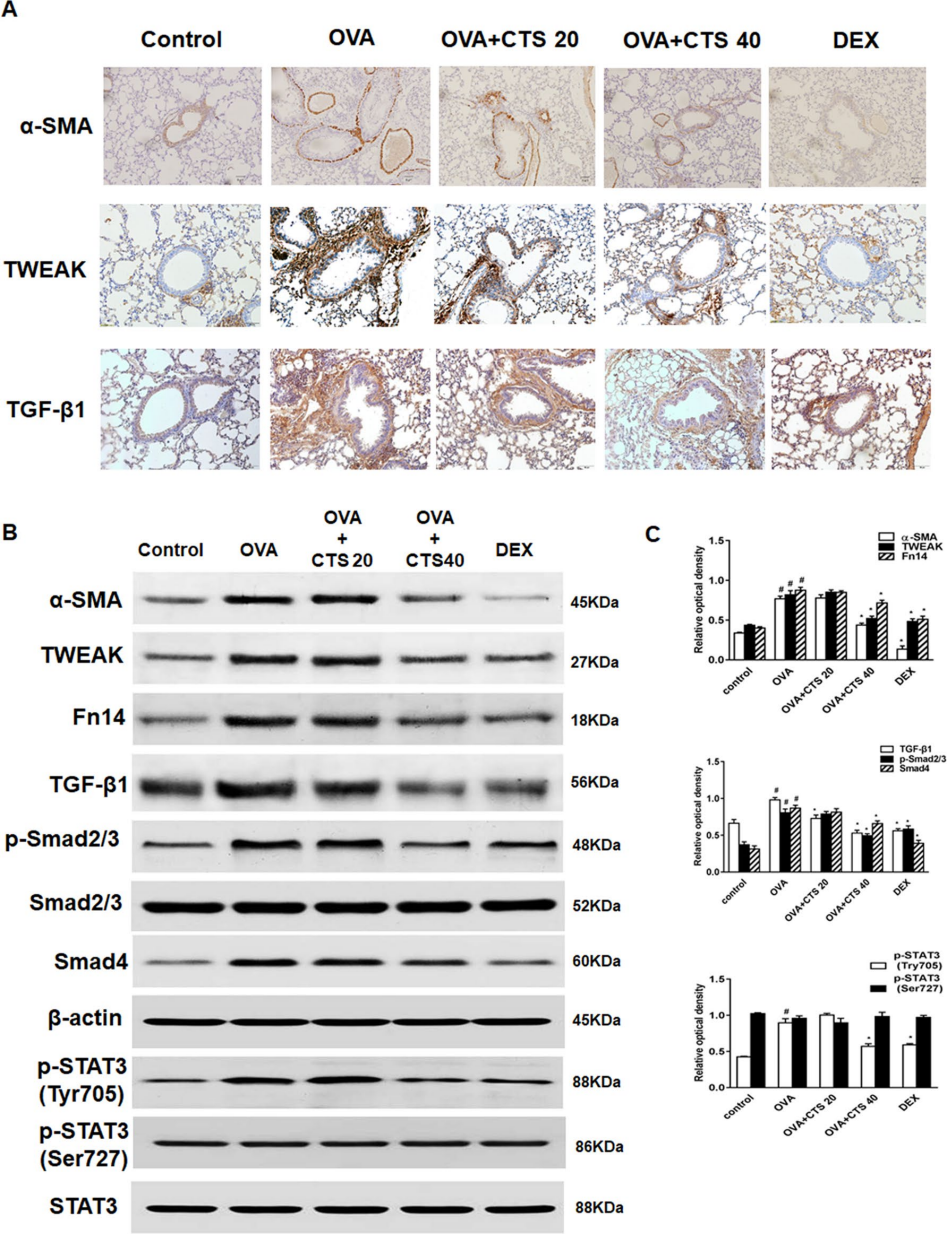
Effect of cryptotanshinone (CTS) on the expression of α-SMA, tumor necrosis factor-like weak inducer of apoptosis (TWEAK), transforming growth factor (TGF-β1), and STAT3 in the lung tissues. **(A)** Immunohistochemical staining was performed to assess the distribution of α-SMA. Magnification was 100×; Scale bar = 200 μm. **(B)** The expressions of α-SMA, TWEAK, Fn14, TGF-β1, Smad4 and phosphorylation of Smad2/3 and STAT3 were analyzed by Western blot. β-actin and STAT3 were used as the standard controls. **(C)** The band intensities of these proteins were expressed as a ratio to β-actin and STAT3. Data were shown as mean ± SEM (n = 8). #P < 0.05 vs. untreated mice; *P < 0.05 vs. OVA-treated mice. Control [phosphate buffered saline (PBS)-inhaled mice administered with PBS], ovalbumin (OVA) (OVA-inhaled mice administered with PBS), OVA+CTS 20/40 (OVA-inhaled mice administered with 20 mg/kg or 40 mg/kg CTS), and dexamethasone (DEX) (OVA-inhaled mice administered with DEX).

Immunohistochemistry and Western blot were used to detect the expressions of α-SMA, TWEAK, and TGF-β1. Representative immunohistochemical staining results for α-SMA were shown in **Figure 3A**. The staining of α-SMA around the airways was increased in the OVA group compared with the control group. CTS and DEX treatment dramatically decreased the areas of α-SMA staining, though the variations in α-SMA staining extent were observed between the CTS and DEX group. These observations were consistent with the results obtained from the Western blot (**Figures 3B, C**). In addition, after immunohistochemical staining, positive PCNA protein-reactive cells were observed in the airway wall of mice in each group, showing as round brown-black stained granules (**Figure S1**). The expression of PCNA in bronchiolar smooth muscle cells in OVA group was higher than that in control group. The expression of PCNA in low dose of CTS group was weaker than that in OVA group. The expression of PCNA in high dose of CTS group and DEX group was lower than that in OVA group.

As shown in **Figure 3A**, the expressions of TWEAK and TGF-β1 increased around the airways in the OVA group, where the number of infiltrated inflammatory cells also increased. However, TWEAK and TGF-β1 expressions were remarkably attenuated by the treatment of CTS and DEX. Western blot results showed that the expressions of TWEAK, Fn14, TGF-β1, Smad4, and phosphorylation of Smad2/3 markedly increased in the OVA mice compared with control mice. High dose of CTS, but not low dose of CTS, significantly reduced these protein expressions (*P* < 0.05) (**Figures 3B, C**). Two phosphorylation sites, Tyr705 and Ser727, of STAT3 were analyzed, and it was found that CTS significantly decreased the phosphorylation of STAT3 (Tyr705), whereas the phosphorylation of STAT3 (Ser727) and total STAT3 protein were not significantly changed (**Figures 3B, C**). These results indicate that CTS can reduce the phosphorylation of STAT3 possibly through TWEAK or TGF-β1 or TWEAK and TGF-β1 crosstalk signaling pathway.

### Effect of Cryptotanshinone on the Cell Apoptosis and Cell Cycle of the TWEAK+TGF-β1-Induced Airway Smooth Muscle Cells

**Figure 4:**
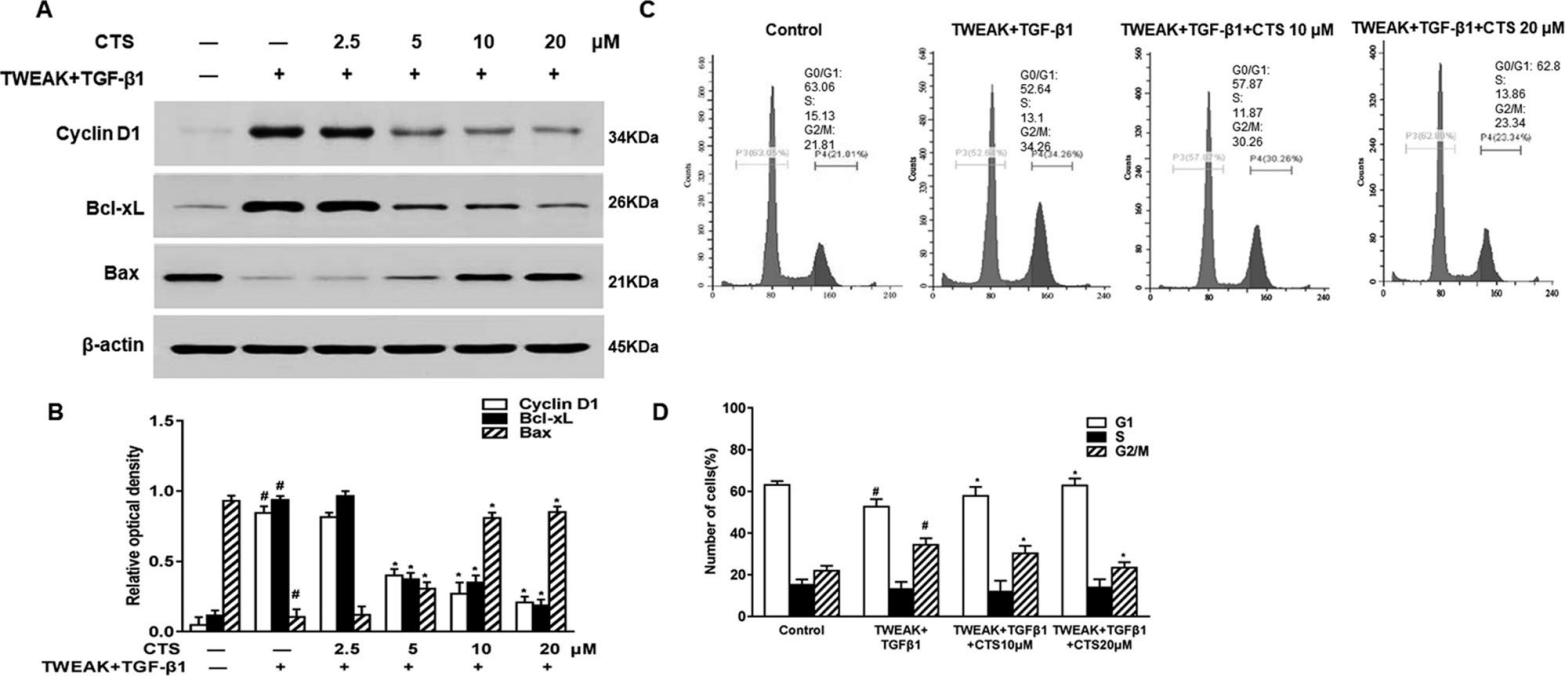
The effect of cryptotanshinone (CTS) on the proliferation of TWEAK + TGF-β1-induced ASMCs is dependent on B-cell lymphoma-extra large (Bcl-xL)/Bax. TWEAK + TGF-β1-induced ASMCs were treated with the indicated concentrations of CTS for 24 h. **(A)** The protein expressions of cyclin D1, Bcl-xL, and Bax were analyzed by Western blot. **(B)** The band intensities of these proteins were quantified in relative to β-actin. **(C)** Representative flow cytometry images of cell cycle analysis of TWEAK+TGF-β1-induced airway smooth muscle cells (ASMCs) after treated with different doses of CTS for 24 h. **(D)** The fractions of ASMCs in G0/G1, S, and G2/M were measured. Data were presented as mean ± SEM (n = 3). #P < 0.05 vs. untreated group; *P < 0.05 vs. TWEAK + TGF-β1 group.

To investigate how CTS can inhibit the TWEAK plus TGF-β1-treated ASMCs proliferation, we detected the expressions of cyclin D1 and its downstream proteins, which have important roles in STAT3-mediated cellular proliferation (Samsonov et al., 2013; Zhang et al., 2014). After TWEAK+TGF-β1 induction, ASMCs were treated with different doses of CTS (0–20 µM) for 24 h, the expressions of cyclin D1, Bcl-xL, and Bax were detected by Western blot. The results showed that the expressions of cyclin D1 and Bcl-xL increased after the stimulation by TWEAK and TGF-β1, while the expression of Bax decreased (**Figures 4A, B**). However, when the TWEAK+TGF-β1-induced ASMCs were treated with CTS, the levels of these proteins decreased in a dose-dependent manner.

To explore the effect of CTS on the TWEAK+TGF-β1-induced ASMCs, these cells were further treated with CTS for 24 h followed by cell cycle analysis. As shown in **Figures 4C, D**, CTS at the doses of 10 and 20 µM arrested cell cycle at G0/G1 phase, and the percentage of cells in G0/G1 phase raised from 52.64 to 62.8%. Accordingly, the percentage of cells in G2/M phase decreased from 34.26 to 23.34%. It was found that CTS increased the number of cells at G1 phase, and decreased the number of cells at G2/M phase. These results indicate that CTS might be an anti-proliferative drug that has a great effect on the cell cycle arrest of the TWEAK+TGF-β1-induced ASMCs.

### Cryptotanshinone Reduces Airway Remodeling Through Tumor Necrosis Factor-Like Weak Inducer of Apoptosis or Transforming Growth Factor Beta 1 Signaling

**Figure 5:**
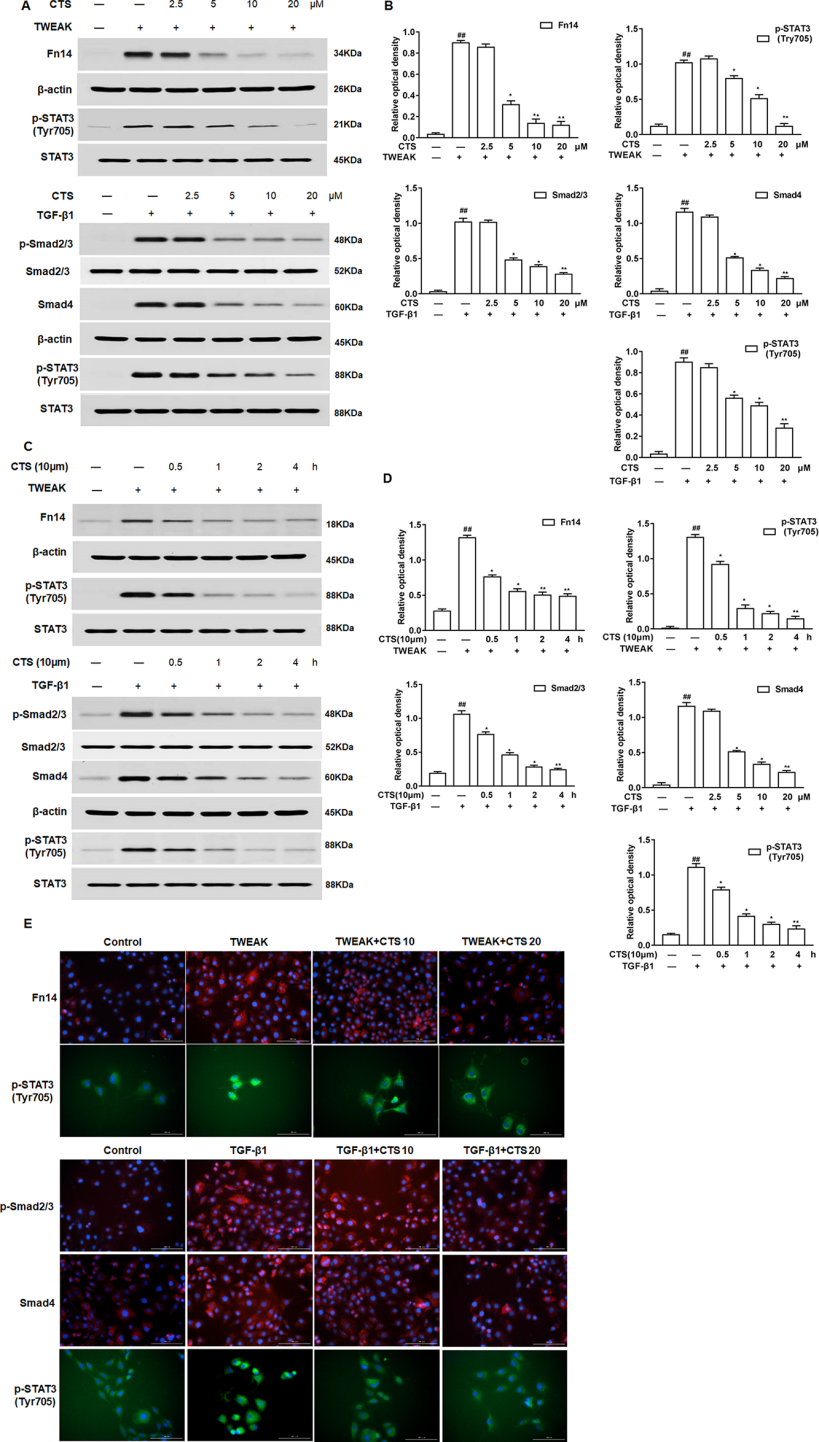
The effect of cryptotanshinone (CTS) on the airway remodeling was through TWEAK/STAT3 and transforming growth factor beta 1 (TGF-β1)/STAT3. Tumor necrosis factor-like weak inducer of apoptosis (TWEAK) or TGF-β1-induced airway smooth muscle cells (ASMCs) were treated with the indicated concentrations of CTS for 24 h. **(A)** and **(C)** The protein expressions of Fn14, p-Smad2/3, Smad4, p-STAT3 (Tyr705), and STAT3 were analyzed by Western blot. **(B)** and **(D)** The band intensities of these proteins were quantified in relative to Smad2/3, β-actin, and STAT3. **(E)** Immunofluorescence staining of TWEAK, TGF-β1, and STAT3 in ASMCs. The magnification was 200×. Fn14 staining (red), p-Smad2/3 staining (red), Smad4 staining (red), p-STAT3 staining (green), and nuclei with 4′,6-diamidino-2-phenylindole (blue) is shown. Data were presented as mean ± SEM (n = 3). ##P < 0.01 vs. untreated group; *P <0.05, **P < 0.01 vs. TWEAK-induced or TGF-β1-induced group.

To verify whether CTS attenuates airway remodeling through TWEAK or TGF-β1 signaling pathway, Western blot was performed after the ASMCs were treated with TWEAK or TGF-β1. Here, we found that CTS significantly decreased the phosphorylation of STAT3 (Tyr705) in a time and dose-dependent manner in ASMCs whatever being treated with TWEAK or TGF-β1 (**Figures 5A–D**). The activation of STAT3 and some other upstream protein kinases of STAT3, including Fn14, p-Smad2/3, and Smad4 was also detected. Results showed that the expressions of these proteins decreased in a time-dependent manner after the treatment with 5–20 µM CTS (**Figures 5A–D**). Immunofluorescence microscopy showed that the intensity of Fn14 and p-STAT3 (Tyr705) staining decreased in the TWEAK-induced ASMCs after treated with CTS. Similarly, the intensity of p-Smad2/3, Smad4, and p-STAT3 staining decreased in the TGF-β1-induced ASMCs after being treated with CTS (**Figure 5E**). These results indicate that CTS might reduce the airway remodeling *via* TWEAK/STAT3 and TGF-β1/STAT3 signaling pathways.

**Figure 6:**
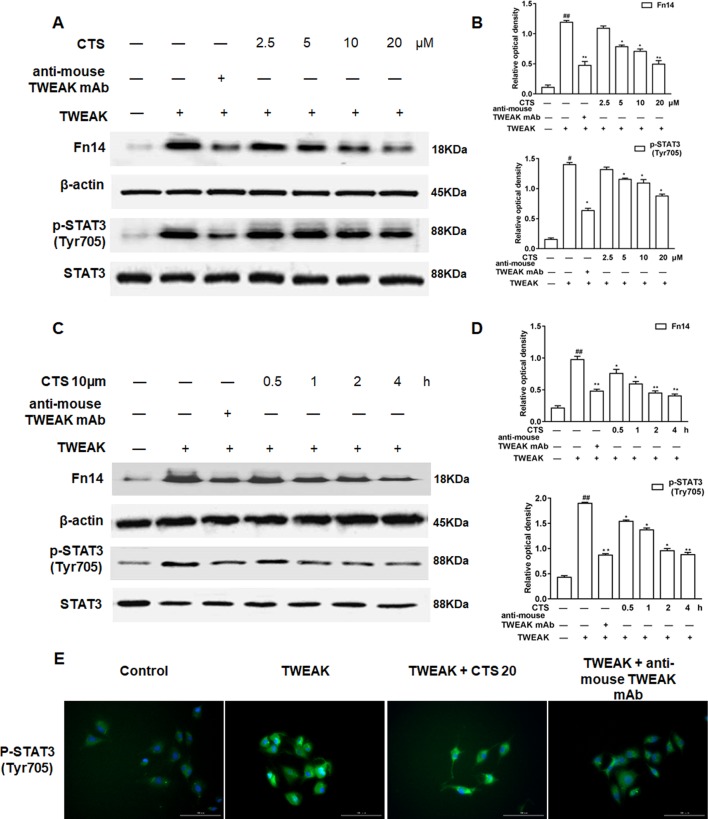
The effect of cryptotanshinone (CTS) on the airway remodeling through tumor necrosis factor-like weak inducer of apoptosis (TWEAK)/STAT3 in TWEAK-induced airway smooth muscle cells (ASMCs). **(A)** Western blot of Fn14, p-STAT3 (Tyr705), and STAT3 in response to the treatment with anti-TWEAK mAB or different doses of CTS in ASMCs. **(C)** Western blot of Fn14, p-STAT3 (Tyr705), and STAT3 under the treatment with anti-TWEAK mAB or 10 μM CTS at different time in ASMCs. **(B** and **D)** Densitometric analysis was presented as the relative ratio of each molecule to Smad2/3, β-actin, and STAT3. Data were presented as mean ± SEM (n = 3). #P < 0.05, ##P < 0.01 vs. untreated group; *P < 0.05, **P < 0.01 vs. TWEAK-induced group. **(E)** The immunofluorescence analysis of p-STAT3 (Tyr705) in ASMCs showed that this protein was mainly expressed in the cytoplasm of ASMCs. Magnification: 200×. As shown in the pictures, p-STAT3 (Tyr705) was stained green and nuclei was stained with 4′,6-diamidino-2-phenylindole showing a blue color.

To further confirm that CTS is closely involved in TWEAK/STAT3 signaling pathway, ASMCs were treated with the anti-TWEAK monoclonal antibody (anti-TWEAK mAB) and the expressions of Fn14, STAT3, and phosphorylation of STAT3 (Tyr705) in ASMCs were analyzed by Western blot. The results showed that the levels of Fn14, p-STAT3 (Tyr705) increased in the ASMCs treated by TWEAK (**Figures 6A–D**). The elevated levels of these proteins significantly decreased by the treatment of anti-TWEAK mAB or different dosages of CTS (5–20 µM) and presented in a time-dependent manner for the treatment with 10 µM CTS. Immunofluorescence showed that TWEAK exposure significantly increased p-STAT3 (Tyr705) in ASMCs (**Figure 6E**). These results indicate that the increases in Fn14 and p-STAT3 (Tyr705) expressions are significantly attenuated by CTS treatment, which has the same effect as anti-TWEAK mAB.

**Figure 7:**
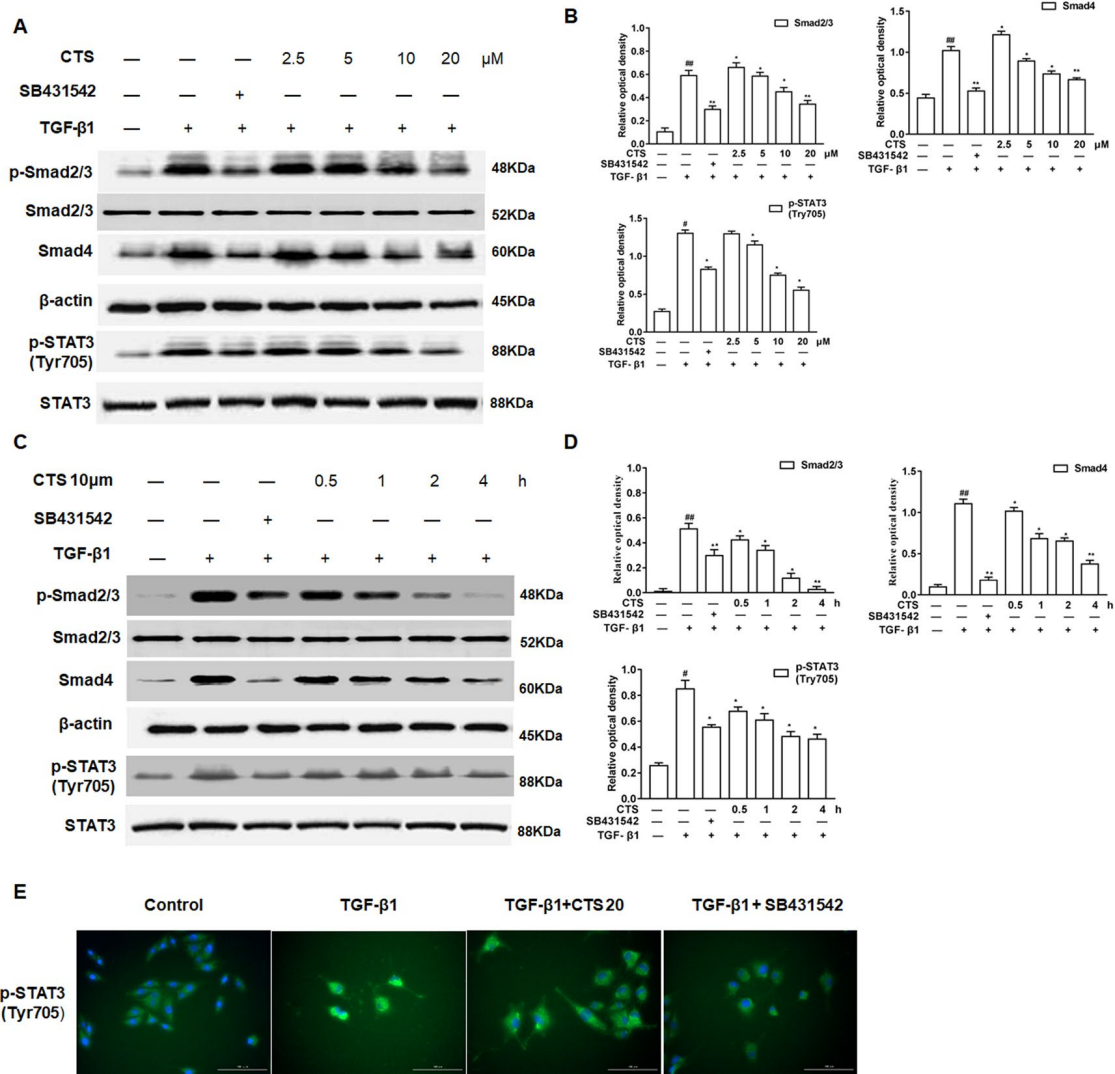
The effect of cryptotanshinone (CTS) on the airway remodeling through transforming growth factor beta 1 (TGF-β1)/STAT3 in TGF-β1-induced airway smooth muscle cells (ASMCs). **(A)** Western blot of TGF-β1, p-Smad2/3, Smad4, p-STAT3 (Tyr705), and STAT3 in response to the treatment with SB431542 or different doses of CTS in ASMCs. **(C)** Western blot of TGF-β1, p-Smad2/3, Smad4, p-STAT3 (Tyr705), and STAT3 under the treatment with SB431542 or 10 μM CTS at different time in ASMCs. **(B)** and **(D)** Densitometric analysis was presented as the relative ratio of each molecule to Smad2/3, β-actin, and STAT3. Data were presented as mean ± SEM (n = 3). #P < 0.05, ##P < 0.01 vs. untreated group; *P <0.05, **P < 0.01 vs. TGF-β1-induced group. **(E)** The immunofluorescence analysis of p-STAT3 (Tyr705) in ASMCs showed that this protein was mainly expressed in the cytoplasm of ASMCs. Magnification: 200×. As shown in the pictures, p-STAT3 (Tyr705) was stained green and nuclei was stained with 4′,6-diamidino-2-phenylindole showing a blue color.

Additionally, to further confirm that CTS is closely involved in TGF-β1/STAT3 signaling pathway, ASMCs were treated with the TGF-β1 inhibitor SB431542 and the phosphorylation of Smad2/3, STAT3 (Tyr705), the expressions of Smad4, and STAT3 in ASMCs were analyzed by Western blot. The results showed that the levels of Smad2/3, Smad4, p-STAT3 (Tyr705) increased in ASMCs after TGF-β1 induction (**Figures 7A–D**). The elevated levels of these proteins significantly decreased by SB431542 or different dosages of CTS (5–20 µM) and presented in a time-dependent manner for the treatment with 10 µM CTS. Immunofluorescence data displayed a similar trend as the Western blot results (**Figure 7E**).

### Cryptotanshinone Inhibits the Activation of STAT3 *via* Tumor Necrosis Factor-Like Weak Inducer of Apoptosis/Transforming Growth Factor Beta 1 Signaling

**Figure 8:**
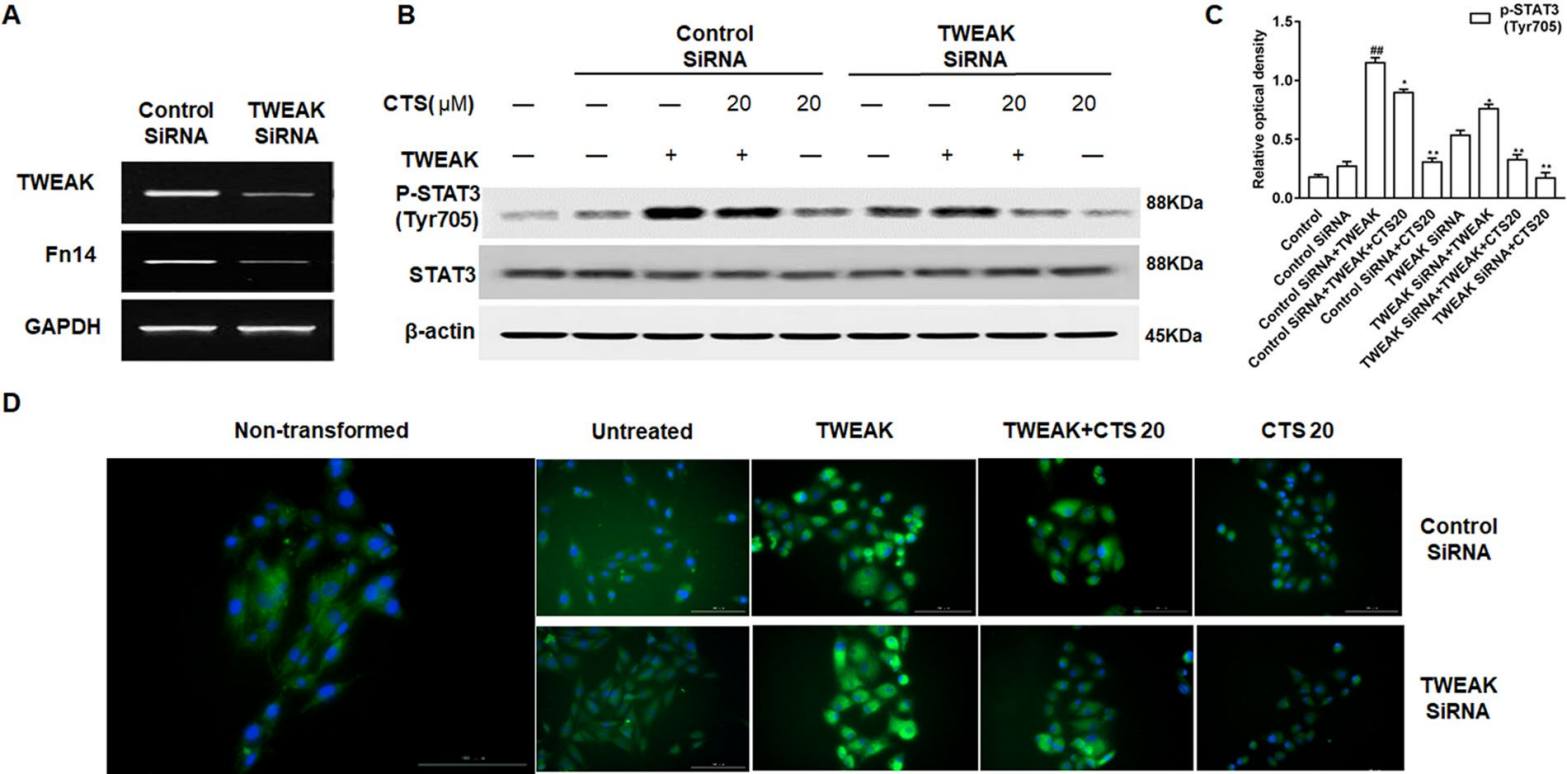
The effect of tumor necrosis factor-like weak inducer of apoptosis (TWEAK) low expression on the phosphorylation of STAT3 in the TWEAK-induced airway smooth muscle cells (ASMCs). **(A)** The expressions of TWEAK and Fn14 in the TWEAK-silenced ASMCs were verified by reverse transcription polymerase chain reaction. **(B)** The protein levels of p-STAT3 (Tyr705) and STAT3 in ASMCs were detected by Western blot after knockdown with TWEAK small interfering RNA. **(C)** Densitometric analysis was presented as the relative ratio of each molecule to Smad2/3, β-actin, and STAT3. Data were presented as mean ± SEM (n = 3). ##P < 0.01 vs. untreated group; *P < 0.05, **P < 0.01 vs. TWEAK-induced group. **(D)** The immunofluorescence analysis of p-STAT3 (Tyr705) in ASMCs showed that this protein was mainly expressed in the cytoplasm of ASMCs. Magnification: 200×. As shown in the pictures, p-STAT3 (Tyr705) was stained green and nuclei was stained with 4′,6-diamidino-2-phenylindole showing a blue color.

To explore if CTS inhibits the activation of STAT3 *via* TWEAK/STAT3 signaling pathway, TWEAK/Fn14 was silenced with TWEAK siRNA in ASMCs (**Figure 8A**) and the expression of STAT3 and phosphorylation of STAT3 were examined by Western blot. In ASMC cells treated with TWEAK siRNA, TWEAK low expression attenuated the phosphorylation of STAT3, and the p-STAT3 (Tyr705) activity was further receded by CTS (**Figures 8B, C**). A significant reduction of p-STAT3 (Tyr705) expression in TWEAK-silenced ASMCs treated with CTS or TWEAK siRNA was also observed by immunofluorescence (**Figure 8D**).

**Figure 9:**
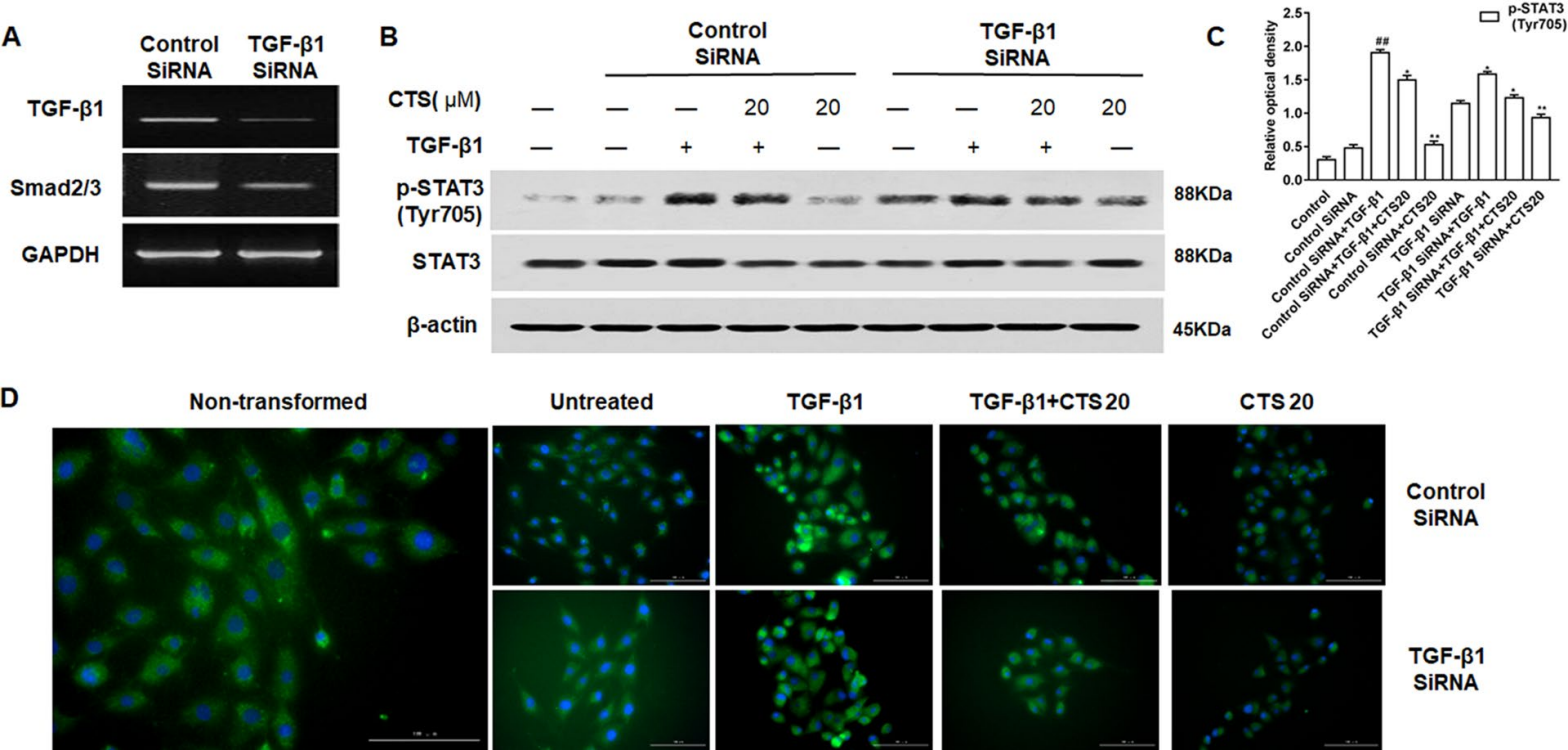
The effect of transforming growth factor beta 1 (TGF-β1) low expression on the phosphorylation of STAT3 in the TGF-β1-induced airway smooth muscle cells (ASMCs). **(A)** The expressions of TGF-β1 and the phosphorylation of Smad2/3 in the TGF-β1-silenced ASMCs were verified by reverse transcription polymerase chain reaction. **(B)** The protein levels of p-STAT3 (Tyr705) and STAT3 in ASMCs were detected by Western blot after knockdown with TGF-β1 small interfering RNA. **(C)** Densitometric analysis was presented as the relative ratio of each molecule to Smad2/3, β-actin and STAT3. Data were presented as mean ± SEM (n = 3). ##P < 0.01 vs. untreated group; *P < 0.05, **P < 0.01 vs. TGF-β1-induced group. **(D)** The immunofluorescence analysis of p-STAT3 (Tyr705) in ASMCs showed that this protein was mainly expressed in the cytoplasm of ASMCs. Magnification: 200×. As shown in the pictures, p-STAT3 (Tyr705) was stained green and nuclei was stained with 4′,6-diamidino-2-phenylindole showing a blue color.

To assess whether CTS is closely involved in TWEAK and TGF-β1 signaling cross talk, TGF-β1 was silenced with TGF-β1 siRNA in ASMCs (**Figure 9A**), and the expression of STAT3 and phosphorylation of STAT3 were examined by Western blot. Knockdown of TGF-β1 markedly attenuated the expression p-STAT3 (Tyr705) in TGF-β1-silenced ASMCs. CTS could further suppress p-STAT3 (Tyr705) in TGF-β1-silenced ASMCs (**Figures 9B–D**). These results indicate that genetically or pharmacologically inhibiting TGF-β1 results in a down-regulation of the immune response and CTS can synergize with TGF-β1 knockdown to inhibit the immune activation of ASMCs (**Figure 9D**).

## Discussion

CTS, a compound purified from *S. miltiorrhiza*, has been reported to exhibit anti-inflammatory effects (Ma et al., 2016) and has a protective effect on lung injury both *in vitro* and *in vivo* (Peng et al., 2016). In the present study, we found that airway inflammation in mice with OVA-induced asthma and the proliferation of ASMCs *in vitro* and the airway remodeling *in vivo* was significantly relieved by CTS. These results suggest that CTS may be a potential therapeutic agent for asthma. Airway inflammation is a main point to of asthma occurrence. Its characteristic is leukocytosis, such as eosinophil infiltration, and excess production of mucus, especially cytotoxic granulosa protein released from eosinophils. In this study, CTS significantly decreased the number of total cells, neutrophils, and eosinophil in the BALFs. Furthermore, it was found that OVA-exposed mice had increased levels of proinflammatory cytokines (TNF-α and IL-1β), Th2 cytokines (IL-4, IL-5, and IL-13), and IgE in the BALFs, inflammatory cells aggregation, goblet cell hyperplasia, mucus production, and collagen deposition. All of these manifestations were typical asthmatic features. However, the increase in these key mediators and allergic symptoms was significantly reduced by the administration of a STAT3 inhibitor, CTS. These results suggest that CTS could reduce OVA-induced asthma inflammation and alleviate asthma symptoms. CTS also inhibited leukocyte migration to endothelial cells by suppressing ICAM-1 and VCAM-1 expression, suggesting the anti-inflammatory role of CTS in allergic response. Additionally, Western blot showed that CTS inhibited the expression of α-SMA and the activation of STAT3. These results suggest that the protective effects of CTS against the OVA-induced airway inflammation and airway hyperreactivity may be due to its ability to inhibit STAT3.

In colorectal cancer cells, CTS inhibited proliferation and growth *via* STAT3 signaling as well (Li et al., 2015). Furthermore, Shin *et al.* reported that CTS inhibited the phosphorylation of STAT3 Tyr705 (Shen et al., 2017). In general, STAT3 has two phosphorylation sites, Tyr705 and Ser727. The Tyr705 site is the main phosphorylation site of STAT3, while Ser727 phosphorylation is considered a secondary event after Tyr705 phosphorylation (Liu et al., 2018). In the present study, the inhibitory effect of CTS on the phosphorylation of STAT3 Tyr705 in ASMCs was observed. However, CTS had no obvious effect on Ser727-phosphorylation. Persistent activation of STAT3 promotes the expressions of target genes (such as cyclin D, Bcl-xL, Bax), and has a very important role in the proliferation of ASMCs. The present study found that CTS not only inhibited cyclin D and Bcl-xL, but also enhanced Bax. During the regulation of cell cycle process, cyclin D1 regulates the checkpoint from G1 phase to S phase (Guo et al., 2013). Our results showed that CTS treatment increased significantly the cell percentage in G1 phase, while decreased the cell percentage in G2/M phase. This result is consistent with previous report (Ke et al., 2017), indicating that CTS treatment can arrest ASMCs in G1 phase. Moreover, PCNA is an intranuclear protein involved in DNA synthesis and plays an important role in cell proliferation. In this study, PCNA expression increased obviously in OVA induced airway remodeling model, indicating that cell proliferation is obvious. CTS could effectively inhibit OVA-induced cell proliferation and reduce airway remodeling.

Although the *in vitro* growth inhibitory effect of CTS on different types of cells has been reported, there have been few reports about the inhibitory effect of CTS on airway inflammation and ASMCs (Teng et al., 2018). We detected the biological effect of CTS on the expressions of some possible upstream regulators of STAT3 signaling, including TWEAK, Fn14, TGF-β1, p-Smad2/3, Smad4, and p-STAT3 (Tyr705) in ASMCs. We have identified that the p-STAT3 (Tyr705) levels are markedly increased in the cell system incubated with TWEAK/TGF-β1, while the level of Fn14 decreased significantly at 5 µM CTS, and they began to decrease at 0.5 h when the dose of CTS was 20 µM. Meanwhile, CTS could decrease the levels of p-Smad2/3 and Smad4 at 24 h when the dose of CTS was 5 µM and at 0.5 h when the dose of CTS was 20 µM. Thus, these data provide direct evidence for the presence of the TWEAK-STAT3 and TGF-β1/STAT3 signaling in TWEAK- or TGF-β1-induced ASMCs, and the immunofluorescence staining also verified the results. Based on these results, we boldly suppose that the attenuation of airway remodeling by CTS may be dependent on TWEAK and TGF-β1 signaling cross-talk.

TWEAK can induce STAT3 phosphorylation at the tyrosine 705 and nuclear translocation, and promote pro-inflammatory cytokine secretion (Park et al., 2017; Wang et al., 2017). In the present study, it was found that, TWEAK-stimulation enhanced the levels of Fn14 and p-STAT3 (Tyr705), which were significantly reduced by CTS or anti-mouse TWEAK mAB. Meanwhile, CTS decreased the expression of Fn14 and phosphorylation of STAT3 Tyr705 protein in a dose- and time-dependent manner. In addition, by silencing TWEAK with siRNA, the inhibition of STAT3 phosphorylation was promoted. Therefore, our findings lend weight to the contention that CTS modulates STAT3 activation through the TWEAK-STAT3 pathway in allergic airway remodeling.

TGF-β1/Smad signaling pathway plays a key role in airway remodeling, which has been widely accepted (Lee et al., 2017). TGF-β1 binds to TGF-β RII to activate TGF-β RI and phosphorylates the downstream receptor-associated R-Smads, including Smad2 and Smad3. Then the p-Smad2 and p-Smad3 form a heteromeric complex with a common Smad, Smad4, and translocate into the nucleus to induce transcription of target genes. Smad4 is a common Smad for TGF-β signaling and plays a critical role for shuttling Smad2/3 and Smad1/5/8 into the nucleus (Chen et al., 2018). It reported that CTS can ameliorate kidney fibrosis and EMT (epithelial mesenchymal transition) by inhibiting the TGF-β1/Smad3 signaling pathway (Chen et al., 2001). This study detected the tyrosine phosphatase activity of STAT3 and the protein levels of Smad2/3 and Smad4 with TGF-β1 inhibitor SB431542 and TGF-β1 siRNA in the TGF-β1-induced ASMCs. We found that the phosphorylation of Smad2/3, expression of Smad4, and the phosphatase activity of STAT3 Tyr705 were decreased by SB431542 and CTS, which showed a dose and time-dependent effect. Similarly, the enhanced phosphatase activity of STAT3 Tyr705 after the stimulation of TGF-β1 was significantly reduced by CTS or siRNA-TGF-β1. Thereby, it is reasonable to assume that CTS can suppress STAT3 by inhibition of TWEAK and TGF-β1 signaling cross talk in the TWEAK- or TGF-β1-induced ASMCs.

**Figure 10:**
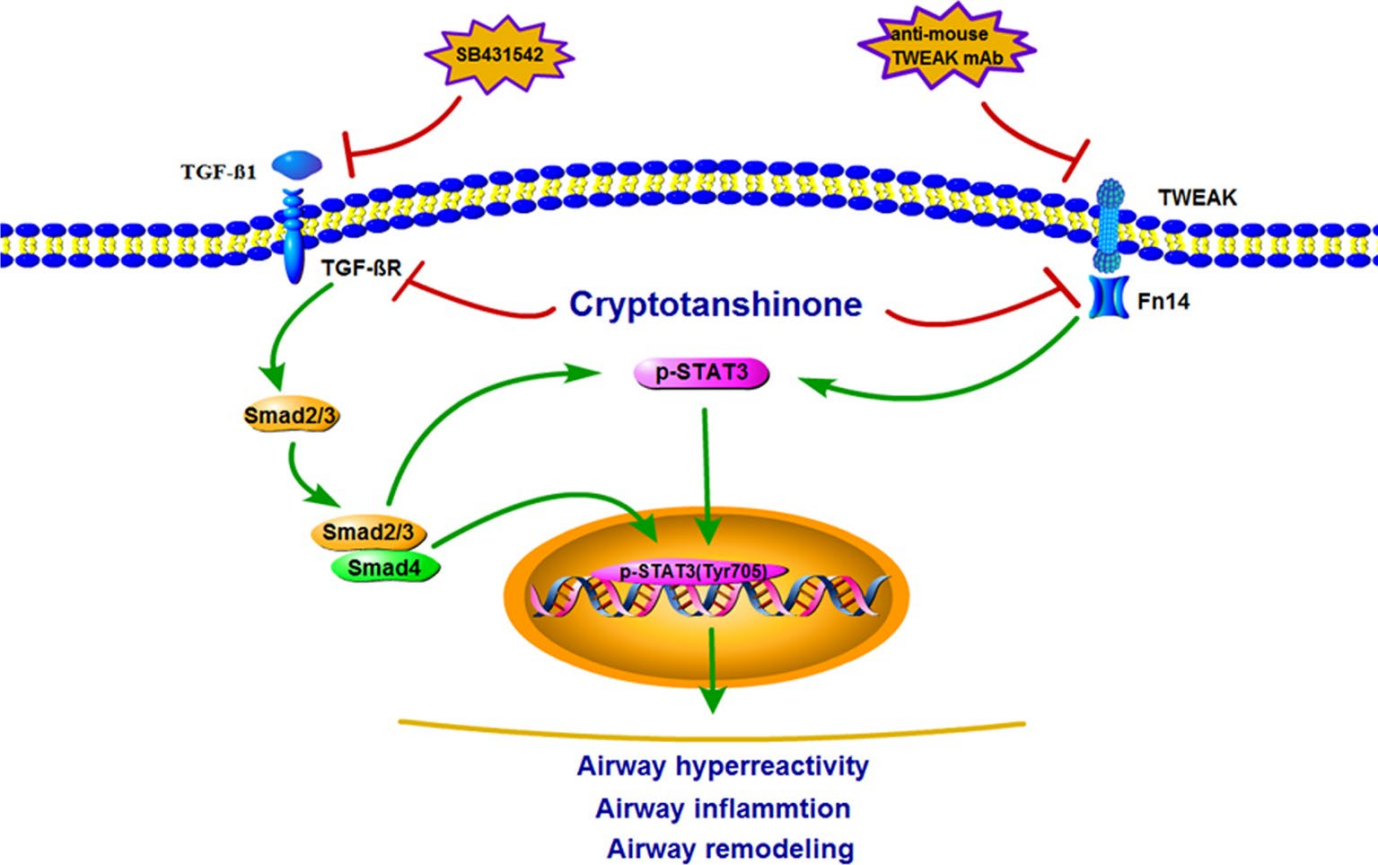
Schematic illustration of the possible molecular mechanism of tumor necrosis factor-like weak inducer of apoptosis (TWEAK)-mediated or transforming growth factor beta 1 (TGF-β1)-mediated airway smooth muscle cells (ASMCs) pro-inflammatory cytokine secretion. TWEAK/Fn14 or TGF-β1/Smad stimulated STAT3 signaling pathways activation in ASMCs, subsequently leading to pro-inflammatory cytokine secretion.

According to the *in vitro* and *in vivo* experimental results, a schematic summary for the mechanisms of inhibitory effect of CTS on STAT3 signaling pathway have been drawn. TWEAK and TGF-β1 could induce STAT3 pathways to activate ASMCs, which plays a vital role in regulating the pro-inflammatory cytokine secretion in airway remodeling (**Figure 10**).

One of the limitations of this study is that the dose-dependent effect of CTS was not investigated in this study. Further studies with multiple doses of CTS are warranted.

In conclusion, the results indicate that CTS may be a potential therapeutic agent of airway remodeling. The mechanism may be related to the suppression of STAT3 and the inhibition of TWEAK and TGF-β1 signaling cross talk.

## Data Availability Statement

The data used to support the findings of this study are available from the corresponding author [GY].

## Ethics Statement

The experiment procedures were approved by the Institutional Animal Care and Use Committee of Yanbian University (Resolution number, 201501022).

## Author Contributions

CW, MZ, YC, JJ, LiL, JL, CX, ZX, YL, and HP performed the experiment. CW, MZ, and YC drafted the manuscript. CW, MZ, YC, JJ, LiL, JL, and CX conducted the data analyses. ZX, YL, and HP searched the literature. LiaL and GY designed the study, guide the experiment and edited the manuscript.

## Funding

This study was supported by the National Natural Science Foundation of China (Grant No. 81560679, 81860729, 81560810 and 81560004).

## Conflict of Interest

The authors declare that the research was conducted in the absence of any commercial or financial relationships that could be construed as a potential conflict of interest.

## Supplementary Material

Figure S1 Effect of CTS on the expression of PCNA in the lung tissues. Immunohistochemical staining was performed to assess the distribution of PCNA. Magnification was 100×; Scale bar = 200 μm. Data were shown as mean ± SEM (n = 8). Control (PBS-inhaled mice administered with PBS), OVA (OVA-inhaled mice administered with PBS), OVA+CTS 20/40 (OVA-inhaled mice administered with 20 mg/kg or 40 mg/kg CTS), and DEX (OVA-inhaled mice administered with DEX).
