# Supplementary figures and images for: Cryptotanshinone Attenuates Airway Remodeling by Inhibiting Crosstalk Between Tumor Necrosis Factor-Like Weak Inducer of Apoptosis and Transforming Growth Factor Beta 1 Signaling Pathways in Asthma
Source: Front Pharmacol. 2019 Nov 11;10:1338. doi: 10.3389/fphar.2019.01338 (PMC6859802; doi:10.3389/fphar.2019.01338)

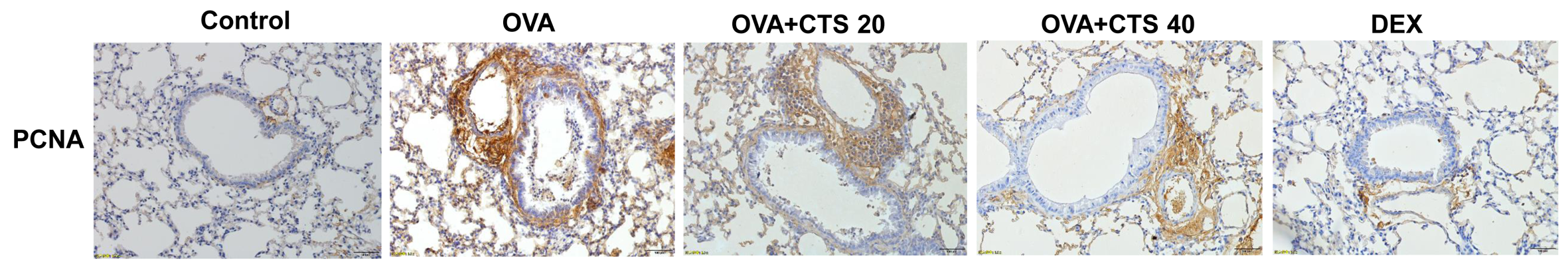

Supplement: Figure S1 — Effect of CTS on the expression of PCNA in the lung tissues. Immunohistochemical staining was performed to assess the distribution of PCNA. Magnification was 100×; Scale bar = 200 μm. Data were shown as mean ± SEM (n = 8). Control (PBS-inhaled mice administered with PBS), OVA (OVA-inhaled mice administered with PBS), OVA+CTS 20/40 (OVA-inhaled mice administered with 20 mg/kg or 40 mg/kg CTS), and DEX (OVA-inhaled mice administered with DEX). [file Image_1.tif]
